# The response of stream ecosystem properties to two size classes of herbivorous minnow species

**DOI:** 10.1002/ece3.10637

**Published:** 2023-10-18

**Authors:** Erika C. Martin

**Affiliations:** ^1^ Department of Biological Sciences Emporia State University Emporia Kansas USA

**Keywords:** algivore, cyprinid, ecosystem, Great Plains, invertivore, mesocosm experiment

## Abstract

Losses in freshwater fish diversity might produce a loss in important ecological services provided by fishes in particular habitats. An important gap in our understanding of ecosystem services by fishes is the influence of individuals from different size classes, which is predicted based on known ontogenetic shifts in metabolic demand and diet. I used 20 experimental stream mesocosms located at Konza Prairie Biological Station (KPBS), KS, USA, to assess the influence of fish size on ecosystem properties. Mesocosms included two macrohabitats: one riffle upstream from one pool filled with consistent pebble and gravel substrate. There were four experimental and one control treatment, each replicated four times (*N* = 20). I used two size classes of central stonerollers (*Campostoma anomalum*) and southern redbelly dace (*Chrosomus erythrogaster*). Five ecosystem properties were assessed: algal filament length (cm), benthic chlorophyll *a* (μg/cm^2^), benthic organic matter (g/m^2^), macroinvertebrate biomass (g/m^2^), and stream metabolism (g O_2_/m^2^/day^−1^). Size structure of fish populations affected some, but not all, ecosystem properties, and these effects were dependent upon species identity. Size structure of both species had effects on algal filament lengths where stonerollers of both size classes reduced algal filaments, but only small redbelly dace kept filaments short. A better understanding of the relationship between these prairie stream minnows and their small stream habitats could be useful to both predict changes in stream properties if species are lost (redbelly dace are a Species In Need of Conservation) or size structure shifts.

## Graphical abstract

Experimental stream mesocosms were used to assess the influence of two prairie steam cyprinids of different size classes on ecosystem properties. Size structure of fish populations affected some, but not all, ecosystem properties, and these effects were dependent upon species identity.

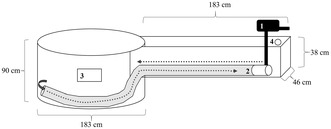

## 1 INTRODUCTION

Freshwater ecosystems contain approximately 10% of all known living species (Winemiller, 2018) including over 14,800 freshwater fish species (Pelayo‐Villamil et al., 2015). Freshwater ecosystems comprise less than 1% of the Earth's surface (Winemiller, 2018), thus housing an inordinate diversity for the given total area. Of particular concern is the projected loss of 4% of all freshwater species (Barbarossa et al., 2021) while simultaneously comprehensive research regarding freshwater fish biodiversity across ecosystems is still needed (Ahmed et al., 2022; Faghihinia et al., 2021). Small bodies of water such as streams are crucial freshwater habitats for a plethora of species, and the need for more targeted research is apparent (Darwall et al., 2018). Specifically, in the Great Plains, fish diversity has been declining due to alterations in land‐use and stream flow patterns (Cross & Moss, 1987; Gido et al., 2010; Taylor, 2010). Losses in freshwater fish diversity will produce a loss in important ecological services provided by fishes in particular habitats (Colin et al., 2018; Radinger et al., 2019). Maintaining biodiversity in small stream systems is particularly critical because they comprise 70% of total stream length (Lowe & Likens, 2005), and these headwater streams begin the downstream transport chain, where inputs into these systems, and the resulting products, travel to downstream locations (Freeman et al., 2007; Vannote et al., 1980).

Efforts by conservation groups such as the Nature Conservancy, a non‐governmental organization (NGO) with stations in every major continent, seek to restore stream ecosystems to support freshwater diversity in Kansas (Tepler & Mehl, 2022). The functional effects of fish species in small freshwater habitats remain poorly understood, even though the research that has been produced generally illustrates that freshwater fish diversity can play an important role in providing unique services to maintain the functioning of these ecosystems (Faghihinia et al., 2021). Studies have shown that freshwater small‐bodied grazing fishes influence stream ecosystem properties (e.g., Bengtson et al., 2008; Bertrand & Gido, 2007; Capps & Flecker, 2013; Evans‐White et al., 2001, 2003; Gelwick & Matthews, 1992; Martin et al., 2016; Power, 1984; Power et al., 1988; Power & Matthews, 1983). The effects of stream fishes on their environment have been assessed by measuring several key ecosystem properties. These environmental factors have been selected for various reasons by past researchers and include, but are not limited to, algal filament length, chlorophyll *a* concentration, benthic organic matter, macroinvertebrate biomass, and primary productivity. These properties are key in understanding resource availability and describing components of the food web. Power and colleagues identified the influence of grazing fishes on the structure of periphyton communities (Power, 1984; Power et al., 1988; Power & Matthews, 1983). Power et al. (1985) showed grazing minnows reduced algal filament lengths in pools, but magnitude and location of effects were altered by the presence of predatory bass. Gelwick and Matthews (1992) measured the effect central stonerollers (*Campostoma anomalum*) had on ecosystem habitat properties and found these grazers reduced organic matter biomass and primary production, and those effects lasted up to 55 days. Subsequent studies have illustrated the potential for stream fish to affect other aspects of ecosystems including changes in benthic invertebrate community structure (Gilinsky, 1984) and nutrient cycling (Flecker et al., 2010; McIntyre et al., 2008; Schmitz, 2008; Vanni, 2002). Gilinsky (1984) stocked bluegill (*Lepomis macrochirus*) in a pond at different densities for one year and reported that fish had a strong impact on diversity and density of some benthic macroinvertebrates, specifically reducing predatory chironomids; however, a seasonal effect, or no effect, was noted for other macroinvertebrate species. Hargrave (2009) found that increasing fish species richness in stream mesocosms was associated with increased mesocosm primary production and that relationship strengthened over time. Martin et al. (2016) assessed fish diversity effects on stream properties by stocking central stonerollers, southern redbelly dace (*Chrosomus erythrogaster)*, and creek chub (*Semotilus atromaculatus*) into experimental mesocosms and found that algal filaments were shorter, floating algal mats smaller, benthic organic matter less abundant, and gross primary production and ecosystem respiration increased in mesocosms subjected to grazer communities compared to no‐fish and grazer‐invertivore communities. Although these studies point toward the potential of herbivorous fishes to influence stream ecosystems, the effects of these fishes on their environment are highly context dependent and are a function of the interaction of many factors, including population size structure. Comparison of size variation of southern redbelly dace and central stonerollers in small Great Plains streams could prove useful because the proportion of juvenile and adult fish often varies substantially in streams spatially and seasonally (Fausch et al., 2002; Hatcher et al., 2017; Hedden & Gido, 2022).

An organism's size affects its physiology and thus its utilization of resources, energetic requirements, and susceptibility to predation (Burress et al., 2013; Kållo et al., 2020; Klemetsen et al., 2003; Walker et al., 2013; Werner & Gilliam, 1984). In order to maximize growth and survival during vulnerable stages of life, fish make ontogenetic shifts in diet (Pilati & Vanni, 2007). Typically, juveniles select proportionally smaller prey while adults have the capability to forage on larger prey (Burress et al., 2013; Nunn et al., 2007). For example, pumpkinseed (*Lepomis gibbosus*) exhibit a strong ontogenetic diet shift; small juveniles feed primarily on soft‐bodied invertebrates while large adults feed on snails (Osenberg et al., 1992). Gizzard shad (*Dorosoma cepedianum*) in western Lake Erie also exhibit ontogenetic diet shifts where juveniles feed on zooplankton and transition to phytoplankton and detritus as they grow (Mundahl, 1988; Price, 1963). Adult central stonerollers are primarily algivorous, particularly preferring diatoms (Power et al., 1988) whereas southern redbelly dace are more omnivorous, consuming algae, or macroinvertebrates (Settles & Hoyt, 1976). Evans‐White et al. (2001) used stable isotopes to show the algivorous central stonerollers obtained no more than 20% of their energy from invertebrates. Additionally, gut contents indicated algae consumption by proportion increased as fish length increased, meaning while these stonerollers are primarily algivorous they become more algivorous with age. In contrast, small (30–50 mm) southern redbelly dace had similar gut contents as larger (> 60 mm total length) individuals (Bertrand & Gido, 2007). Ontogenetic diet shifts are species dependent, and research on ontogenetic dietary changes in fish is relatively common (Sånchez‐Hernåndez et al., 2019). In contrast, the potential ecosystem consequences of an ontogenetic dietary shift in a species have not been extensively studied (Benoit et al., 2021), particularly regarding small‐bodied cyprinids (Braga et al., 2012; Villeger et al., 2017).

Energetic requirements for juvenile fishes are often more intense than for adults of the same species. An equation by Brett (1962) used to estimate metabolic demand shows that 21 grams of adult fish will have roughly 50% of the metabolic demand of juvenile fish. In general, the energetic requirements per individual is a function of respiration, waste, and growth (Peters, 1983), where an increase in organism mass corresponds to declines in specific metabolic rates (all other variables equal, Clarke & Johnston, 1999; Peters, 1983). Peters (1983) used data from previous studies to illustrate the linear relationship, with a slope less than one, of body mass (kg) to a standard metabolic rate (Watts), and showing most organisms adhere to this predictable pattern, if variations in thermal regulation (i.e., endotherm or ectotherm) are taken into account. This relationship also predicts that larger individuals have a proportionally lower metabolic rate than smaller individuals (see also Winberg, 1956). Using the metabolic scaling equation of Peters (1983), metabolism for a single fish weighing 20 grams is 47.44% of 20 fish weighing 1 g each. This illustrates the potential for small fish to have disproportionately large impacts on stream properties when compared to larger fish, assuming all other factors are equal.

Southern redbelly dace and central stonerollers (hereafter referred to as redbelly dace and stonerollers) are two small cyprinids that impact structural (algal filament length, organic matter) and functional (stream metabolism) properties of streams (e.g., Bengtson et al., 2008; Bertrand & Gido, 2007; Bonjour et al., 2020; Gelwick & Matthews, 1992; Martin et al., 2016; Power & Matthews, 1983). Few studies have directly analyzed the relationship of fish size and their influence on stream ecosystem properties. While Fowler and Taber (1985) found that stonerollers have the potential to consume up to 27% of their body weight daily, size was unfortunately not reported specifically for the individuals collected to analyze food habits, but a separate study within the same paper looking at feeding periodicity reported standard lengths of 30–74 mm. These two minnows are some of the most abundant species in small order (<3rd) pristine Great Plains prairie streams, with the size and structure of their populations varying within and across years (Franssen et al., 2006; Martin et al., 2013; Pennock et al., 2018).

The purpose of this study was to use stonerollers and redbelly dace to assess the influence ontogenetic size differences within a species may have on that species' impact to ecosystem properties. For the study described herein, I measured five stream properties where prior work (described above) has indicated fish do have an effect: algal filament length (cm), benthic chlorophyll *a* (μg/cm^2^), benthic organic matter (g/m^2^), macroinvertebrate biomass (g/m^2^), and stream metabolism (g O_2_/m^2^/day^−1^). While there is substantial work demonstrating that fish do indeed affect stream properties and that fish diet preferences and/or habitat usage changes as the fish grows, fish effects on stream properties as they grow is not well studied. Using work by Brett (1962), it is reasonable to predict a 2 × reduction in ecosystem effects for larger bodied individuals in comparison to small‐bodied individuals of the same species, particularly for ecosystem factors most directly associated with metabolic needs (e.g., food). A better understanding of the relationship between these prairie stream minnows and their small stream habitats could be useful to developing future conservation assessments. This understanding is particularly important to redbelly dace populations, a Species In Need of Conservation, as designated by the Kansas Department of Wildlife and Parks (2009).

## 2 METHODS

### 2.1 Mesocosm design and preparation

**FIGURE 1:**
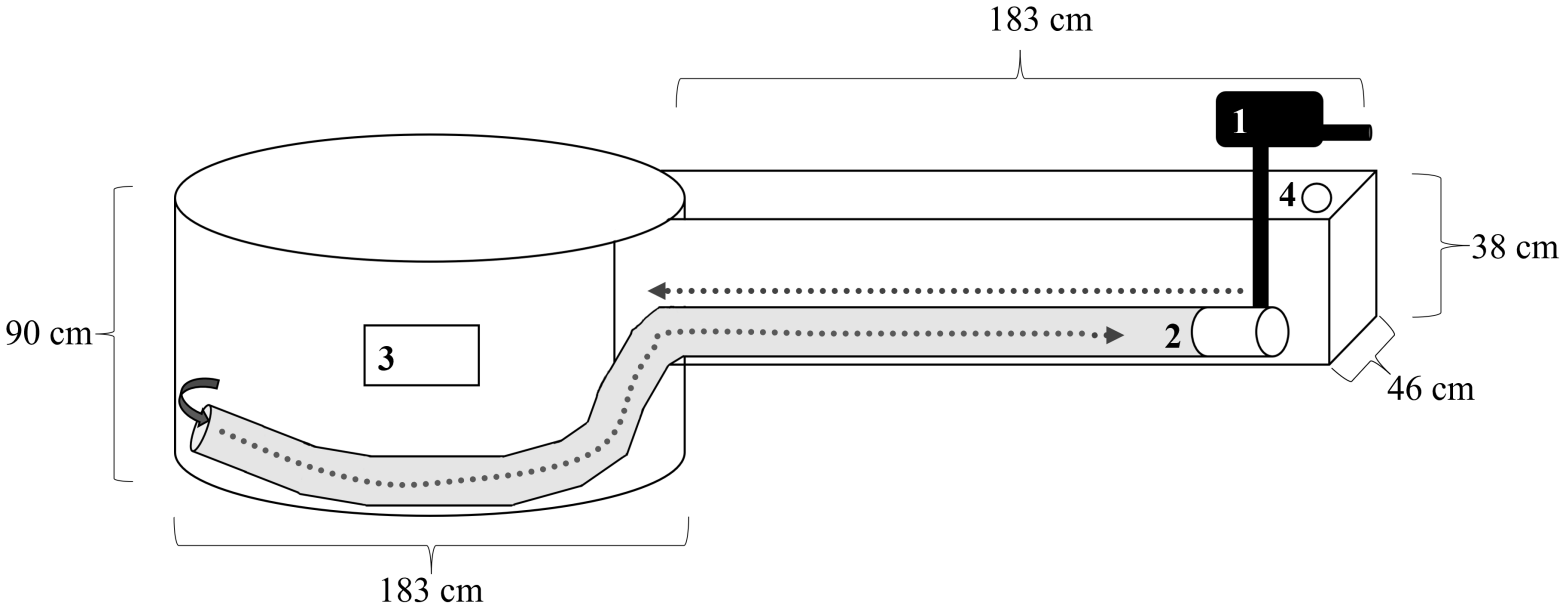
Diagram of one experimental mesocosm which houses a trolling motor (1) in the riffle that directs flow into a pool. Water is drawn up from the pool through a corrugated pipe (2). Observations can be made looking down into the water or through a viewing window (3). There is an aerial PVC dripline that drips fresh groundwater into the riffle near the trolling motor. When the entire mesocosm is filled, excess water is released through a drain (4).

I used 20 experimental stream mesocosms located on Konza Prairie Biological Station (KPBS), KS, USA (also described in Martin et al., 2016; Matthews et al., 2006; but see Figure 1), to test how fish size influence stream ecosystems. The experiment ran for 30 days (October 1, 2012, to October 31, 2012). Mesocosms included two macrohabitats: one riffle (area = 0.8 m^2^) upstream from one pool (area = 2.5 m^2^) filled with consistent pebble and gravel substrate (mean area 40 cm^2^). Flow was generated by a trolling motor pulling water through a 15.2 cm diameter solid black corrugated plastic drainage pipe connecting the pool to the riffle. Prior to the start of the experiment, streams were drained of any incidental water (e.g., rainwater) and left dry for four full weeks. Next, I scoured and washed the substrate and inside of each mesocosm with a pressure washer until all surfaces were clean, after which streams were drained and left to dry for several days. Finally, mesocosms were filled with spring water using an underground PVC pipe system from the same springs that naturally fill nearby Kings Creek. Mesocosms take 2–3 days to fill completely, and once all mesocosms were filled, trolling motors were turned on and mesh baskets filled with cleaned substrate with open tops were buried in the substrate.

### 2.2 Mesocosm homogenization

Allowing mesocosms to be fully dry for long periods of time and then pressure washing the substrate and walls promoted homogeneity among units. Furthermore, prior to beginning the experiment, flow and depth were measured using a Marsh‐McBirney flowmeter and meter stick along three transects in each riffle (*n* = 9) and at along four transects in each pool (*n* = 19) within each reach for roughly 10 measurements per m^2^ for each habitat, and a mean flow and depth were calculated for each macrohabitat. Based on an analysis of variance (ANOVA), there was no evidence for significant differences among habitat types across mesocosms for flow (i.e., all riffles were similar to each other and all pools were similar to each other; *F*_6,42_ = 0.97, *p* = .46) or depth (*F*_6_,_42_ = 0.18, *p* = .98).

### 2.3 Experimental design

There were four experimental treatments and one control (no fish) treatment, each replicated four times (*N* = 20 mesocosms). Experimental treatments were additions of fish based the two most abundant herbivorous species (stonerollers and redbelly dace) in Kings Creek, the stream located on the KPBS, and one control treatment. All fish (*N*_total_ = 2336) were collected from nearby Kings Creek and stocked on September 28, 2012, and given 2 days to acclimate. Each species was represented by two size classes, creating four fish treatments: small stonerollers (mean total length (TL) = 56 mm ± 3.9 mm standard deviation (SD), mean weight (*W*) = 0.76 g ± 0.17 g), large stonerollers (TL = 81 mm ± 2.7 mm, *W* = 2.35 g ± 0.24 g), small redbelly dace (TL = 31 mm ± 1.6 mm, *W* = 0.13 g ± 0.01 g), and large redbelly dace (TL = 53 mm ± 1.1 mm, *W* = 0.65 g ± 0.09 g). Weights of fishes were calculated from standard length (SL) values using the standard length–weight equation *W* = *aL* ^*b*^ where *a* = 0.0037 and *b* = 3.08 (K. Gido, unpublished data). Cutoff lengths for juveniles versus adults (stonerollers = 65 mm and redbelly dace = 55 mm) were based on published accounts of length at maturity for both species (Edwards, 1997; Hubbs & Cooper, 1936; Martin et al., 2013; Settles & Hoyt, 1978). All fish were measured (total length) prior to stocking mesocosms. Fish biomass was 15 (±3) g/m^2^, 16 (±1.3), for juvenile and adult stonerollers, respectively, and 29 (±12.4) and 24 (±1.7) for juvenile and adult redbelly dace, respectively. The total biomass of these fishes varies significantly in the Kings Creek system, and our biomass reflects the highest end of this natural variation (Martin et al., 2016). Fish deaths and TL of dead were recorded and replaced with similar sized fish during the acclimation period. The fifth treatment served as a control with no fish added to the streams.

### 2.4 Response variables

Five variables were selected as they comprise the basic ecosystem functioning and food web dynamics in streams and are based on extensive work in natural and experimental streams (algal filament length [AFL; cm], benthic chlorophyll *a* [μg/cm^2^], benthic organic matter [g/m^2^], macroinvertebrate biomass [g/m^2^], and stream metabolism [g O_2_/m^2^/day^−1^], Bertrand & Gido, 2007; Evans‐White et al., 2001; Martin et al., 2016; Murdock et al., 2011). These ecosystem properties were measured in pools at the end of the experiment. To quantify structural properties of the periphyton, AFL were measured in centimeters using a meter stick along four transects in each pool (*n* = 19) within each mesocosm for 10 measurements per m^2^ for each pool. Filament lengths were measured as the length of the longest filament attached to a pebble that occurred on the transect point. To assess benthic chlorophyll *a*, macroinvertebrates, and organic matter, the three 10 cm^2^ mesh baskets were filled with pebbles and buried in each pool prior to the start of the experiment were collected. One basket was removed at the end of the experiment for chlorophyll *a* analysis; three pebbles were collected from the basket, and chlorophyll *a* was extracted where the concentration of chlorophyll *a* was corrected for cross‐sectional area of pebbles (see Bertrand & Gido, 2007; Sartory & Grobbelaar, 1984 for detailed methods on chlorophyll *a* extraction). The second basket was removed to quantify coarse and fine benthic organic matter (CBOM and FBOM, respectively) analysis. The basket was placed into 8 L of water from the mesocosm and the substrate vigorously stirred by hand. Next, a 500 mL subsample of the slurry was collected. The subsample was preserved using 10% formalin, taken to the laboratory, filtered through two mesh sizes (coarse [1 mm mesh filter] and fine [GF/F 47 mm microfiber filter]), dried at 60°C, weighed, ashed at 450°C, and re‐weighed to determine ash‐free dry mass (AFDM) (Wallace et al., 2007). AFDM was extrapolated using the surface area of the basket. Macroinvertebrates were sampled by a similar procedure to organic matter, where the third basket was placed in 8 L of water from the mesocosm and vigorously stirred by hand to release attached macroinvertebrates. Here, the resulting slurry was elutriated to separate inorganic substrate from organic matter and poured through a sieve (250 μm mesh) to capture macroinvertebrates. Samples were preserved in 10% formalin and taken to the laboratory where invertebrates were counted and identified to order or family (Merritt et al., 2008; Thorp & Covich, 2001). Chironomids were initially classified as Tanypodinae or non‐Tanypodinae; however, because Tanypodinae chironomids constituted <1% of sample biomass, all chironomids were combined into a single group. Lengths of macroinvertebrates were estimated to calculate biomass for each macrohabitat using standard length–mass relationships (Benke, 1984). Macroinvertebrate biomass was calculated by dividing the total biomass of the sample by the surface area of the basket. Finally, whole stream metabolism (gross primary production [GPP]) was measured based on fluctuations in dissolved oxygen content (g O_2_/m^−2^/day^−1^) of the water measured every 10 min over a 24 h period using a YSI ProODO optic dissolved oxygen sensor. The sensor was deployed in the same location in each mesocosm pool near the observation window (Figure 1). These rates were corrected for variation in temperature, dissolved oxygen saturation, light, atmospheric pressure, and stream mesocosm morphology based on the single‐station modeling technique outlined in Riley and Dodds (2013) and Dodds et al. (2013). In short, this method uses a standard equation (Marzolf et al., 1994) to predict dissolved oxygen concentration. Modeled dissolved oxygen (see Martin et al., 2016 for model example) is compared to observed dissolved oxygen using the Solver function in Microsoft Excel (version 2007; Microsoft Corporation, Redmond), which uses a Newton search method to minimize the sum of squares of error between modeled and observed values by changing the basic rates of GPP, ER, and gas transfer coefficient (*k*).

### 2.5 Data analysis

Variables were centered and standardized. Differences among treatments for each of the five response variables were tested by performing analysis of variance (ANOVA) tests to assess treatment effects (*α* = 0.05) comparing juvenile stonerollers, adult stonerollers, and the control. Separate ANOVAs compared juvenile redbelly dace, adult redbelly dace, and control. If ANOVA models were significant, treatments were compared using the post‐hoc analysis Tukey's Honestly Significant Difference (HSD) adjustment. In addition, due to the low power of the experiment, Cohen's *d* estimates of effect size (Cohen, 1988) were calculated. All analyses and graphs used program R (version 4.3.1, R Core Team, 2023) and libraries ggplot2 (Wickham, 2016), car (Fox & Weisberg, 2011), lsmeans (Lenth, 2016), and effsize (Torchiano, 2017).

## 3 RESULTS

### 3.1 Overall

Mean AFL ranged from zero to 24 cm. Mean chlorophyll *a* concentration ranged from 0.62 to 1.1 μg/cm^2^. Mean CBOM ranged from 0.16 to 0.86 g/m^2^. Mean FBOM ranged from 0.95 to 1.65 g/m^2^. Mean macroinvertebrate biomass ranged from 4.23 to 27.49 g/m^2^. The primary invertebrates in the mesocosms were daphnia, chironomids, ostracods, and dipterans. Mean stream metabolism ranged from 6.4 to 6.8 g O_2_/m^2^/day^−1^.

### 3.2 Stonerollers

ANOVA models found no significant treatment effects for chlorophyll *a*, CBOM, FBOM, macroinvertebrate biomass, or stream metabolism. Analyses did indicate a treatment effect on AFL (*F*_2,9_ = 6.9, *p* = .0152). Tukey HSD post‐hoc analysis identified a difference between fish treatments and the control (*p* < .027), where both small and large stonerollers had similar effects, cropping filament lengths very short in comparison to the control.

**FIGURE 2:**
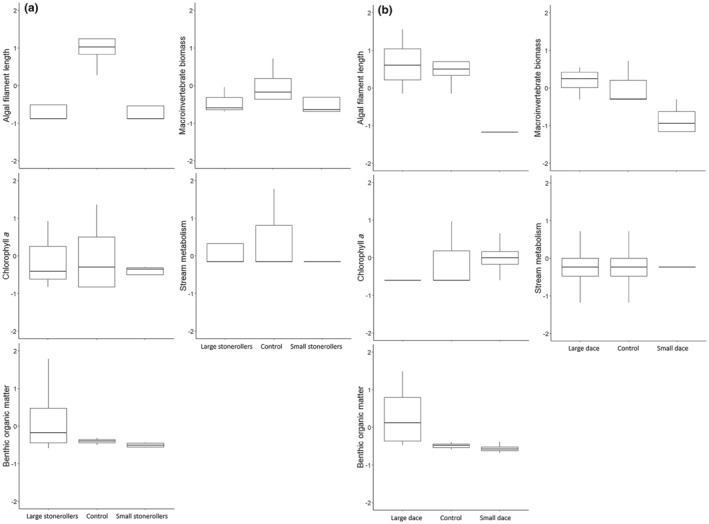
Boxplots for all response variables for (a) central stonerollers and (b) southern redbelly dace. Data were centered and standardized.

Cohen's *d* calculations found large effect sizes within four of the response variables but not stream metabolism (Table 1). For AFL, like the ANOVA results, analysis found a large effect size where both size classes of stonerollers reduced filament length compared to the control (Figure 2). While other response variables did indicate an effect size – some of them large – most of the 95% confidence intervals overlapped zero (Table 1). Chlorophyll *a* effects were seen for all stoneroller treatment comparisons, and small stonerollers had the lowest variation where control treatments had the highest variation. Additionally, moderate and small effect sizes were found between the control and both the small and large stoneroller treatments, respectively, where the fish treatments had lower median values (Figure 2). Benthic organic matter in large stoneroller treatments had the highest variation. Large effect sizes were calculated when comparing the small stoneroller treatments to both the control and large stoneroller treatments, where small stoneroller treatments had the lowest benthic organic matter (Figure 2). Macroinvertebrate biomass complements AFL results, where the fish treatments had lower macroinvertebrate biomass estimates than the control, and the effect sizes were small for large stonerollers and moderate for small stonerollers, where small stonerollers had a lower macroinvertebrate biomass estimates than large stonerollers (Figure 2). For stream metabolism, a small effect size was indicated between large stonerollers and the control, and a moderate effect size comparing the two stoneroller size classes. The median was not different among the three treatments, but the control had the highest variation, followed by large stonerollers (Figure 2).

**TABLE 1:** Results from Cohen's *d* effect size calculations and the 95% confidence interval.

Response variable	Comparison	Cohen's *d*	95% CI
Stonerollers			
Algal filament length	**Small stoneroller–Control**	2.40***	**0.13 to 4.66**
	Large stoneroller–Control	−2.22***	−4.42 to 0.02
	Small stoneroller–Large stoneroller	0.05	−1.68 to 1.78
Chlorophyll *a*	Small stoneroller–Control	0.58**	−1.18 to 2.35
	Large stoneroller–Control	0.41*	−1.34 to 2.16
	Small stoneroller–Large stoneroller	0.94***	−0.88 to 2.77
Benthic organic matter	Small stoneroller–Control	0.81***	−0.99 to 2.61
	Large stoneroller–Control	−0.70	−1.80 to 1.66
	Small stoneroller–Large stoneroller	0.94***	−0.89 to 2.76
Macroinvertebrate biomass	Small stoneroller–Control	0.65**	−1.12 to 2.43
	Large stoneroller–Control	0.30*	−1.44 to 2.04
	Small stoneroller–Large stoneroller	0.59**	−1.18 to 2.36
Stream metabolism	Small stoneroller–Control	0.00	−1.73 to 1.73
	Large stoneroller–Control	0.37*	−1.38 to 2.11
	Small stoneroller–Large stoneroller	0.71**	−1.08 to 2.49
Dace			
Algal filament length	**Small dace–Control**	4.16***	**1.08 to 7.24**
	Large dace–Control	0.19	−1.55 to 1.92
	**Small dace–Large dace**	3.54***	**0.77 to 6.31**
Chlorophyll *a*	Small dace–Control	0.52**	−1.24 to 2.28
	Large dace–Control	−1.10***	−2.95 to 0.76
	Small dace–Large dace	−1.69***	−3.71 to 0.33
Benthic organic matter	Small dace–Control	0.77**	−1.03 to 2.56
	Large dace–Control	0.05	−1.68 to 1.78
	Small dace–Large dace	1.34***	−0.57 to 3.25
Macroinvertebrate biomass	Small dace–Control	1.52***	−0.45 to 3.48
	Large dace–Control	−0.49*	−2.24 to 1.27
	**Small dace–Large dace**	2.62*	**0.26 to 4.98**
Stream metabolism	Small dace–Control	−0.62**	−2.39 to 1.15
	Large dace–Control	0.00	−1.73 to 1.73
	Small dace–Large dace	−0.62**	−2.39 to 1.15

*Note*: Asterisks indicate effect sizes, which were defined as: * small (0.20–0.49), ** medium (0.50–0.79), and *** large (≥0.80). Bolded comparisons indicate a notable effect size and 95% CI that does not overlap zero.

### 3.3 Redbelly dace

ANOVA models found no significant treatment effects for chlorophyll *a*, CBOM, FBOM, macroinvertebrate biomass, or stream metabolism. Analyses did indicate a treatment effect on algal filament length (*F*_2,9_ = 14.4, p = .0016). Tukey HSD post‐hoc analysis identified a difference between small dace treatments compared to large dace and control treatments (*p* < .004), where small dace cropped filaments short, but large dace did not.

Cohen's *d* indicated moderate to large effects for all variables (Table 1). For AFL, like the ANOVA results, analysis found a large effect size where small dace had much shorter filaments than both the large dace and control treatments (Figure 2). Macroinvertebrate biomass had a small effect between size class treatments, a large effect size between small dace and the control, and a small effect between large dace and the control. The general trend here was large dace had the highest macroinvertebrate biomass values, followed by the control, and finally small dace had lowest macroinvertebrate biomass estimates (Figure 2). While the other three response variables did indicate an effect size, again, most of the 95% confidence intervals overlapped zero (Table 1). For chlorophyll *a*, large effect sizes were calculated between large dace and the control, and large dace and small dace. A medium effect size was indicated between small dace and the control. Generally, chlorophyll *a* values were lowest for large dace and highest for small dace (Figure 2). A moderate and large effect size was seen comparing small dace to the control and to large dace for benthic organic matter, respectively, where small dace had the lowest values (Figure 2). For stream metabolism, a moderate effect was found comparing small dace to the control and small dace to large dace treatments. The median was not different among the three treatments, but small dace had the least variation (Figure 2).

## 4 DISCUSSION

The ranges of the five ecosystem response variables fall well within expectations and complement other studies in similar mesocosm systems (Martin et al., 2016; Parr et al., 2020), which provides support that comparisons of this experiment to others in mesocosm systems is reasonable. Parametric analyses found a significant effect of treatments on AFL only, where both stoneroller treatments lowered AFL compared to the control but only small dace lowered AFL and these results were corroborated with effect size calculations. The prediction of a 2 × reduction in ecosystem effects of large redbelly dace compared to small redbelly dace was evident (exact difference was 1.6×). However, this same differential magnitude was not observed when comparing stoneroller size classes. The differences in the redbelly dace treatments might be due to changes in dietary preferences and not metabolic demand, and there was a small effect in macroinvertebrate biomass between the small and large dace treatments. While the data presented herein has low sample sizes, future work might explore on ontogenetic diet shifts of dace with a focus on intraspecific competition. It would be of interest to explore this across seasons, especially when young of year individuals are in high densities.

Cropping of AFL by adult stonerollers but not adult redbelly dace has been observed in a previous study in this system (Martin et al., 2016), and when cropping of algal filaments does not occur the filaments reach the stream surface and amalgamate into large floating algal mats which can influence primary production under the water surface, alter habitat and food resources for macroinvertebrates and fishes, and potentially influence public use of the stream (Martin et al., 2016; Tan et al., 2021). Martin et al. (2016) looked at the effect of fish community diversity on stream properties using stonerollers, dace, and creek chub (*Semotilus atromaculatus*) and found that even if fish biomass remains constant, the reduction of stonerollers can facilitate algal mat formation. Other studies have shown both stonerollers (Power et al., 1985, 1988; Power & Matthews, 1983; Veach et al., 2018) and redbelly dace (Bertrand & Gido, 2007) to have large negative effects on AFL. However, these latter studies did not directly address size differences. Results from this study provide further evidence complementing previous studies that demonstrated stonerollers do not have ontogenetic diet shifts, primarily feeding on algae regardless of size.

None of the other response variables were found to be significantly affected by treatment. While numerous effect sizes are indicated, the confidence intervals overlap zero. Thus, we are unable to discern if the effect of fish is big or small from these data; the sample size is too small to estimate it well. However, it is worth discussion and leads to some interesting questions for future work. Algal filament length was altered, but not total benthic chlorophyll *a* concentration, though chlorophyll *a* exhibited high variation in control treatments. This suggests that regardless of the structural height of the benthic algal community, the total photosynthetic output remains stable. It would be instructive to run the same, or similar, experiment and allow algal growth to progress for several months to facilitate floating algal mat development from uncropped filaments and follow the flux of chlorophyll *a* from the benthos to the surface. Moreover, the benthic algal community might be changing due to direct and indirect effects of fishes (e.g., Brooks & Dodson, 1965; Grutter et al., 2022), and more detailed quantification of the algal community would be interesting. If the fish are selectively feeding on particular species or groups, this potential reduction of certain photosynthesizers might help explain the reduced variance in fish treatments. Benthic organic matter was ubiquitous across treatments, but most variable in large fish treatments for both species. Organic matter enters the systems through natural pathways, such as falling leaves which are broken down by weathering or invertebrate shredders. Results assessing organic matter and its response to fishes have been mixed. Murdock et al. (2011) found redbelly dace decreased total benthic organic matter in experimental mesocosms early after a flood in spring, but these effects diminished over time. An experimental mesocosm study by Bertrand and Gido (2007) found that under more stable conditions in summer, redbelly dace increased fine particulate organic matter. Previous studies have shown stonerollers influence benthic organisms through bioturbation (Adámek & Maršálek, 2013) by scraping algae off substrates (Fowler & Taber, 1985; McNeely, 1987). A thorough examination of the effects of stonerollers and mussels (*Amblema plicata* and *Actinonaias ligamentina*) on benthic organic matter and chlorophyll *a* found positive associations with grazing fish biomass and benthic organic matter and chlorophyll *a* (Parr et al., 2020). The relationship between animals and organic matter is complex and highly context dependent.

Macroinvertebrate biomass was lowered by small dace, and while non‐significant and small or moderate, stonerollers of both sizes might reduce macroinvertebrate biomass as well. Gido et al. (2010) have found grazers reduce chironomids. A similar study in this same mesocosm system found stonerollers increased macroinvertebrate biomass, but the addition of dace reduced macroinvertebrate biomass in pools – but the opposite pattern was observed in riffles (Martin et al., 2016). Future studies should incorporate different habitats, and habitat use by fishes as a potential explanatory variable as smaller fish prefer shallower stream habitats in natural systems (Martin et al., 2013; Power, 1987). In a previous mesocosm study, adult dace gut contents were found to be more similar to riffle communities than pools, suggesting they drift feed from riffles (Kohler et al., 2011). Previous research has shown that these juvenile fishes use different stream habitats than the adult fishes, where juveniles prefer shallower areas and cover and are more abundant in small stream (<3rd order) headwaters than in mainstem (>3rd order, Hedden & Gido, 2022; Martin et al., 2013). Therefore, streams may experience a reduction in structural properties such as algal filament lengths and organic matter concentration where juvenile fishes congregate. A mesocosm study by Vaughn et al. (1993) demonstrated that stonerollers strongly affect secondary production of two invertebrate grazers (crayfish [*Orconectes virilis*] and snails [*Physella virgata*]). While no crayfish or snails were present in the mesocosms of my study, they certainly are found co‐occurring with stonerollers and redbelly dace in natural systems.

Stream metabolism was not different among treatments, but variance was least in both treatments of small fish. No differences among treatments could be a result of lower power, but might be explained by the scale of measurements, stream metabolism integrates processes across pools *and* riffles. If fish are spending the majority of their time in one habitat, productivity in the other habitat might increase, especially given the recirculating flow (i.e., fish graze in pools, reducing filaments, but high‐nutrient wastewater is pulled from pools and recirculated over riffles), exaggerating their impact on whole stream metabolism.

Fish population dynamics are often cyclical, especially in regard to reproductive or colonization/migration timing. Post‐spawning events, when eggs have hatched and fry and juveniles are abundant, might have a particularly large impact on stream properties due to sudden consumption of resources. Likewise, fish effects on stream properties might be exacerbated when small headwater stream reaches are re‐wetted and colonized by large individuals when algal and macroinvertebrate biomass might already be low (Murdock et al., 2011). This study suggests that size structure could be an important consideration when quantifying a species' effects on stream properties and demonstrates several potential avenues for future research. Understanding the ontogenetic shifts of grazing fishes in these experimental mesocosms is one cog in the machine of understanding the complex spatial and temporal effects these species might have on natural stream systems.

## AUTHOR CONTRIBUTIONS

**Erika C. Martin:** Conceptualization (equal); data curation (equal); formal analysis (equal); investigation (equal); methodology (equal); writing – original draft (equal); writing – review and editing (equal).

## CONFLICT OF INTEREST STATEMENT

The author has no conflict of interest to disclose.

## Data Availability

Data and materials supporting the results and analyses presented in this paper are available at the Konza Prairie LTER KNZ Data Catalog http://lter.konza.ksu.edu/data.
